# Epidermal growth factor receptor signaling modulates postoperative pain and inflammatory responses

**DOI:** 10.3389/fimmu.2026.1793260

**Published:** 2026-05-08

**Authors:** Annet Kyomuhangi

**Affiliations:** 1 Molecular Modeling and Biopharmaceutical Center, College of Pharmacy, University of Kentucky, Lexington, KY, United States; 2 Department of Pharmaceutical Sciences, College of Pharmacy, University of Kentucky, Lexington, KY, United States

**Keywords:** EGFR and chemokines, EGFR and cytokines, EGFR and opioid receptors, EGFR and pain, EGFR ligands and pain, EGFR/PI3K/Akt/mTOR and postoperative pain

## Abstract

Postoperative pain (POP) arises from the activation and dysregulation of nociceptive pathways following tissue injury. Although it plays a protective role by signaling potential harm and preventing further damage, POP can become maladaptive when inflammatory and neural processes intensify or prolong pain signaling. Surgical trauma triggers an immune response that sensitizes nociceptors, lowering the threshold for pain. Simultaneously, acute inflammation skews the balance between pain facilitation and inhibition in favor of pain facilitation, resulting in central sensitization and subsequent chronic postoperative pain. Emerging evidence indicates that inhibition of the EGFR signaling pathway may offer a novel therapeutic approach for pain management. This is supported by preclinical and clinical data showing robust analgesic and anti-inflammatory effects in chronic pain contexts. Furthermore, the EGFR-activated PI3K-Akt-mTOR pathway has been implicated in rodent models of postoperative pain. Despite these promising findings, conclusive data regarding the analgesic efficacy of this pathway in postoperative recovery remains limited. EGFR inhibition may mitigate the substantial adverse effects of current pain medicines, thereby addressing a critical unmet need in clinical pain management. This review explores the role of EGFR pathways in pain and inflammation, with an emphasis on its interaction with other receptors and how these interactions influence tissue survival and inflammatory processes.

## 1 Introduction

Postoperative pain arises primarily from sensitization of peripheral nociceptors, a process characterized by lowered activation thresholds, amplified responses, and expansion of receptive fields (1–4). Surgical incision, despite being sterile, triggers a substantial immune response through the release of damage-associated molecular patterns (DAMPs). These endogenous danger signals activate pattern recognition receptors on resident immune cells, initiating a cascade that clears cellular debris and initiates tissue repair (5). As inflammation progresses, mast cells, neutrophils, and monocytes infiltrate the wound bed (6–8), releasing growth factors that promote re-epithelialization, angiogenesis, and collagen organization. These immune cells also secrete cytokines and chemokines that regulate cell survival, differentiation, and communication within the wound environment (9). The cumulative effect is a complex inflammatory milieu, an “inflammatory soup” composed of lipids, cytokines, chemokines, neuropeptides, prostaglandins, protons, histamine, growth factors, and bradykinin (10, 11). Nociceptors express a wide range of receptors that detect these mediators, and prolonged exposure induces peripheral sensitization (12, 13) through enhanced surface expression and activity of ion channels such as voltage-gated sodium channels (VGSCs), transient receptor potential (TRP) channels, and acid-sensing ion channels (ASICs) (14).

Although most postoperative pain management strategies target classical inflammatory pathways, several drugs approved for unrelated indications, including antibiotics, antiepileptics, antidepressants, and anticancer agents, have demonstrated analgesic properties (15). Among these, epidermal growth factor receptor (EGFR) inhibitors have emerged as particularly promising. Agents such as erlotinib (16, 17), afatinib (18–20), and cetuximab (21, 22) have produced notable pain relief and improved quality of life in both oncologic and non-oncologic settings. These effects are increasingly attributed to the role of EGFR signaling in both peripheral and central sensitization through multiple downstream pathways (23–27). Supporting this mechanistic framework, EGFR and its ligands are upregulated in dorsal root ganglion (DRG) neurons after injury (28, 29), and EGFR activation consistently evokes pain-facilitating responses in animal models (26, 30–35). Beyond its role in nociceptive signaling, EGFR regulates several inflammatory processes (36–40). Its inhibition upregulates IL-10 production (41), and suppresses acute severe inflammatory cascades (42). In chronic diseases such as collagen-induced arthritis and rheumatoid arthritis, inhibiting EGFR suppresses the expression of inflammatory mediators, preventing joint damage (43, 44). These anti-inflammatory processes are largely coordinated through EGFR crosstalk with other receptors including cytokine receptors and G-protein coupled receptors (GPCRs) (45–48).

Although the antinociceptive effects of EGFR-targeted signaling have been demonstrated in several chronic pain models, its role in postoperative pain and the associated inflammatory response remains insufficiently defined. This gap is significant, as postoperative pain is driven by a robust inflammatory milieu characterized by the release of mediators that bind to receptors on nociceptors, leading to sensitization. In parallel, EGFR activation within nociceptors can directly modulate ion channel activity and neurotransmitter release, thereby promoting neuronal hyperexcitability. This review aims to synthesize current evidence on EGFR signaling and its interactions with key receptors to establish a conceptual framework for understanding its role in inflammation and pain modulation. We propose that EGFR functions as a central signaling hub that integrates inflammatory and neuronal signals, thereby influencing both peripheral sensitization and central plasticity. Consequently, EGFR represents a context-dependent therapeutic target: its modulation may attenuate peripheral inflammation while simultaneously limiting maladaptive synaptic plasticity that contributes to chronic postoperative pain.

## 2 Methods

### 2.1 Literature identification and selection

This narrative review was designed to synthesize current knowledge on the role of EGFR signaling in postoperative pain and inflammation and its interaction with other receptors. Relevant literature was identified through targeted searches of PubMed, Web of Science and Embase using combinations of keywords including “EGFR and Pain”, “EGFR/PI3K/Akt/mTOR in postoperative pain”, “EGFR and chemokine transactivation”, “EGFR and TNFα and inflammation” “IL-1β and EGFR in inflammation”, “IL-6 and EGFR and inflammation”, “PGE_2_-EGFR transactivation”, “EGFR and IL-10 signaling”, “EGFR and Opioid receptors” and “EGFR transactivation and TGFβ”.

### 2.2 Study scope and inclusion criteria

The literature search prioritized peer-reviewed experimental, translational, and clinical studies that investigated EGFR in the context of inflammation, chronic pain, and/or postoperative pain. Both *in vitro* and *in vivo* studies were included as well as clinical studies. Mechanistic studies examining EGFR activation through ligand-dependent or ligand-independent pathways were emphasized.

### 2.3 Exclusion criteria

Studies were excluded if they lacked relevance to inflammatory or pain-related signaling, focused exclusively on oncologic outcomes without accompanying inflammatory or nociceptive endpoints, or failed to provide mechanistic insight into EGFR signaling pathways. Articles relying solely on network pharmacology approaches without supporting mechanistic validation were also excluded. In addition, non-peer-reviewed publications, retracted articles, conference abstracts, and studies limited to rodent pain behavior analyses without accompanying cellular or molecular data were excluded. Finally, studies focused exclusively on other members of the EGF receptor family were not considered.

### 2.4 Data extraction and synthesis

Research paper abstracts and where possible actual data were manually screened based on key signaling pathways investigated, animal experiments performed, and cellular and molecular biology experiments. Key signaling pathways, animal models, cellular models and test compounds were extracted from each article. Attention was given to EGFR-mediated regulation of PGE_2_, cytokines, chemokines, matrix metalloproteinases and pain.

### 2.5 Analytical approach

The literature was interpreted using a mechanistic and integrative framework, focusing on how EGFR intersects with inflammatory mediators to modulate pain and inflammation. This approach was chosen to provide conceptual clarity and biological insight rather than exhaustive discussion of literature.

## 3 EGFR expression and ligands in the CNS

EGFR is broadly expressed throughout the CNS with substantial expression reported in the cortex (49–58), hippocampus (59–64), spinal cord (65–68) and nociceptive neurons (33, 69, 70). In neurons, EGFR expression is enriched at presynaptic (71) and non-synaptic sites (63), suggesting that EGFR regulates synaptic connectivity since its loss uncouples axon branching from synaptic formation (71). In contrast, EGFR expression in astrocytes (55, 61, 66–68, 72, 73) and microglial (37, 73–75) is highly dynamic and is markedly upregulated in response to injury and inflammatory stimuli. EGFR ligands, including epidermal growth factor (EGF), transforming growth factor-α (TGF-α) and heparin-binding EGF-like growth factor (HB-EGF), are synthesized by both neurons and glial cells and can be released in an activity-dependent manner (76–81), enabling context-specific activation of EGFR signaling within CNS circuits.

Ligand diversity, localization and distribution confer context specificity to EGFR signaling. For example, HB-EGF expression is strongly induced in the CNS after hypoxic or injury-related stress, with elevated HB-EGF mRNA and protein detected in cortical neurons under hypoxic conditions and increasing expression during ischemic or seizure-related activity, suggesting dynamic regulation in response to neuronal stress and activity (76). Astrocytes also upregulate HB-EGF production during early neuroinflammatory stages in experimental autoimmune encephalomyelitis, highlighting injury and inflammation-driven ligand induction in glia (82). Moreover, neuronal activity such as glutamatergic neurotransmission promotes the shedding and release of HB-EGF from neurons, indicating that synaptic activity can locally modulate EGF-family ligand availability and potentially activate nearby EGFR (83). These features allow EGFR to function as a sensor of both physiological activity and pathological stress through context-dependent ligand expression and release.

### 3.1 Canonical EGFR signaling pathways in sensory neurons

Rodent models of chronic pain report EGFR presence in dorsal root ganglia (DRG) (28) and NF200^+^, CGRP^+^, and IB4^+^ neurons (70), supporting nociceptor EGFR signaling capacity. Studies of EGFR signaling in sensory neurons provide direct evidence that EGFR ligand binding activates canonical downstream pathways classically associated with receptor tyrosine kinases. In cultured mouse DRG neurons, stimulation with EGF rapidly induces phosphorylation of ERK1/2 and STAT3 (84), suggesting engagement of MAPK and JAK/STAT axes downstream of EGFR receptor activation. Functional studies further demonstrate that EGF enhances acid-evoked ASIC currents in rat DRG neurons via an EGFR-dependent mechanism requiring ERK activation, but not JNK or p38 signaling (85). *In vivo*, EGFR expression and activation increase in DRG neurons in chronic compression neuropathic pain models (26, 28), where EGFR promotes mTOR signaling and pain hypersensitivity, consistent with canonical PI3K/Akt/mTOR outputs downstream of EGFR (26, 28). These findings establish that sensory neurons possess functional EGFR and that classic EGFR pathways such as MAPK/ERK, STAT3 and Akt/mTOR can be engaged in this cell type.

Mechanistically, EGFR’s activated cytoplasmic domain binds and phosphorylates PLCγ, a key step enabling IP_3_-mediated Ca^+2^ release from intracellular stores (86). In neuronal contexts, studies in *Caenorhabditis elegans* demonstrate that neuronal EGFR (LET-23) engages PLCγ to regulate synaptic vesicle release and behavior (87), demonstrating that EGFR-PLCγ signaling can regulate synaptic plasticity and neurotransmitter release. Additionally, EGFR signaling can transactivate the tropomyosin receptor kinase B(TrkB) in neural precursor cells with associated phosphorylation at the PLCγ signaling site, supporting a role for EGFR-linked PLCγ signaling in neural lineages (88).

### 3.2 Non-canonical EGFR signaling mechanisms

Non-canonical EGFR signaling mechanisms exist in the CNS and in inflammatory settings. GPCRs can induce ligand-independent transactivation of EGFR via intracellular kinases, leading to receptor phosphorylation and downstream signal engagement without classical ligand binding (50, 89). In innate immune pathways, EGFR has been shown to interact with Toll-like receptors, where EGFR kinase activity is required for TLR3 phosphorylation, and antiviral signaling (90), and for TLR7 and TLR9 tyrosine phosphorylation (91, 92) that enables adaptor recruitment and inflammatory gene induction. However, overexpression of EGFR during CNS injury, confers neuroprotection (93–95) via activation of the EGFR/STAT3 pathway. These non-canonical pathways are particularly relevant in contexts of injury and inflammation, where EGFR integrates signals from cytokines, DAMPs and cellular stress to modulate immune responses.

## 4 EGFR signaling mechanisms in inflammation and pain

**Figure 1:**
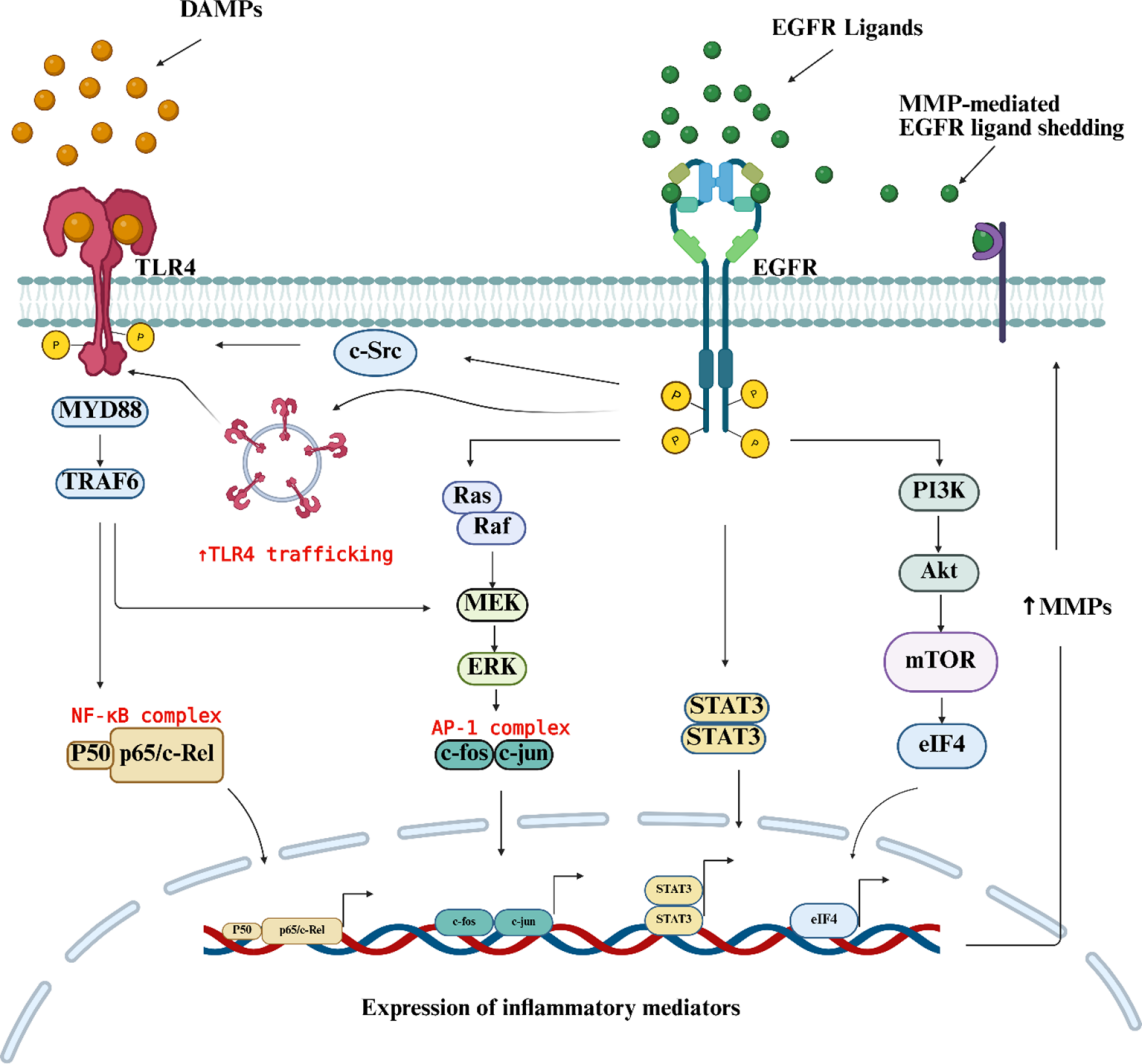
EGFR and TLR4 signaling mechanisms in peri-incision tissue. Surgical tissue injury triggers the release of DAMPs, which bind to TLR4 and activate downstream transcription factors, including AP-1 and NF-κB. This activation promotes the expression of inflammatory mediators, such as cytokines, which in turn induce MMPs that activate EGFR ligands. Consequently, EGFR signaling leads to src-dependent phosphorylation of TLR4 and facilitates its endosomal trafficking. Created in https://BioRender.com.

EGFR is a receptor tyrosine kinase that regulates cell survival, proliferation, differentiation, and inflammation. It is activated by a family of structurally related ligands, including epidermal growth factor (EGF), transforming growth factor-α (TGF-α), heparin-binding EGF-like growth factor (HB-EGF), amphiregulin (AREG), epiregulin (EREG), betacellulin(BTC), and epigen (96, 97). Canonical EGFR signaling is initiated when ligand binding induces receptor dimerization, followed by autophosphorylation of tyrosine residues within the cytoplasmic tail (98). These phosphorylated motifs recruit adaptor and effector proteins that activate intracellular signaling cascades, including the MAPK/ERK (37, 75, 99), PI3K/Akt/mTOR (26, 100, 101), JAK/STAT (102), and NF-κB (103–105) pathways leading to inflammation and pain (**Figure 1**) (106–108). However, EGFR signaling is not uniformly proinflammatory, as it’s activation in CNS hemorrhage is neuroprotective and promotes an M2 microglial phenotype (93, 95). Furthermore, reduced EGFR signaling can exacerbate degenerative conditions such as osteoarthritis, where EGFR appears to support cartilage homeostasis (109, 110). These findings suggest that EGFR senses tissue microenvironment cues and activates relevant pathways based on these cues.

Beyond its established roles in tissue biology and oncogenesis, EGFR signaling modulates nociceptor excitability and epigenetic reprogramming of nociceptive neurons (111). Accumulating evidence identifies EGFR as a mediator of pain across neuropathic (112), inflammatory (113, 114), visceral (115), and cancer-related contexts (**Table 1**) (35, 116, 117 ). In rodent models of peripheral nerve injury, enhanced EGFR expression and activation correlates with mechanical allodynia and thermal hyperalgesia, whereas pharmacologic or genetic disruption of EGFR attenuates these pain behaviors (28, 111, 118). Clinical evidence further supports these findings with case series, prospective observational studies, and a randomized proof-of-concept trial reporting rapid and sustained analgesia in patients with refractory neuropathic pain treated with EGFR inhibitors (**Table 1**) (21, 119–124 ). Importantly, analgesic responses occurred independent of tumor regression (21), indicating a direct effect on pain pathways rather than a secondary consequence of reduced tumor burden.

**Table 1:** EGFR antagonism alleviates pain: preclinical and clinical evidence.

Study type	Model/population	Pain context	EGFR inhibitors	Key findings	Reference(s)
Preclinical (Rats)	Lumbar DRGs Chronic compression	Neuropathic pain	EGFR targeting siRNA Gefitinib	↓cold allodynia ↓mechanical allodynia ↓thermal hyperalgesia	(28)
Preclinical (Rats)	Cancer-induced bone pain	Bone pain, morphine tolerance	AG1478	↓EGFR expression ↓morphine tolerance ↓microglia activation	(117)
Preclinical (Mice)	Sciatic Nerve Injury model	Neuropathic pain	EGFR^inh-III^ Erlotinib EGFR targeting siRNA	↓mechanical allodynia ↓thermal hyperalgesia ↓neuronal excitation	(35, 111)
Preclinical (*In vitro*)	Spinal Cord Astrocytes	Central Sensitization	AG1478	ATP-induced PGE_2_ release requires EGFR transactivation	(114)
Clinical (case series)	Cancer patients	Neuropathic cancer pain	Erlotinib, gefitinib, cetuximab	↑pain relief	(120, 121, 124)
Clinical (observational)	Refractory cancer pain	Neuropathic pain	Panitumumab	↑pain relief	(112)
Clinical (RCT, proof-of-concept)	Neuropathic pain patients	Neuropathic pain	Cetuximab	↑pain relief	(119)
Preclinical (Rats)	Visceral pain models	Visceral hypersensitivity	PD153035	↓serotonin transporter	(115)
Preclinical (Mice)	Osteoarthritis models	Degenerative joint pain	Gefitinib, Erlotinib	↑cartilage damage	(109, 110)
Translational	Complete Freud’s Adjuvant (CFA), formalin and carrageenan induced pain; Spared nerve injury (SNI), Chronic sciatic nerve constriction (CCI); human cohorts of chronic pain	Inflammatory and Neuropathic Pain	Lapatinib, Gefitinib, AG1478	↓allodynia ↓hyperalgesia	(26)

In inflamed or injured tissues, EGFR activation is driven by both classical ligand and receptor transactivation mechanisms. Inflammatory stimuli such as mechanical stress and GPCR ligands, promote ligand-independent EGFR activation through intracellular kinases or ligand-dependent activation via metalloproteinase-dependent shedding of membrane-anchored EGFR ligands (38, 97)(see details below).

Collectively, EGFR structure and signaling design enable the receptor to function as a dynamic sensor of injury and inflammatory stimuli engaging relevant signaling pathways to coordinate cell survival, tissue repair, and immune response mechanisms. However, the same signaling mechanisms that support tissue survival can, when sustained or dysregulated, drive chronic inflammation and pain. Therefore, understanding EGFR temporal and spatial regulation in inflammatory context is essential for the rational design of therapeutic strategies aimed at enhancing tissue survival and blocking excessive or chronic inflammatory response.

### 4.1 EGFR ligands in pain

EGFR ligands EGF, EREG and AREG are implicated in both neuropathic and inflammatory pain states. EGFR ligand availability is regulated by matrix proteinases(MMPs) (125), activated by TLRs and GCPRs promoting ectodomain shedding of membrane-bound ligands. Epiregulin (EREG) has emerged as a potent modulator of nociception. Even without overt tissue injury, intrathecal EREG induces robust thermal and mechanical hypersensitivity (26). Human genetic studies show that loss-of-function EREG variants correlates with reduced chronic pain severity but increased acute pain perception (26, 33), suggesting that EREG may exert proinflammatory effects during acute immune responses, contributing to inflammation and acute pain. In line with this argument, EREG expression is upregulated early during inflammation, and promotes the expression of additional growth factors, which in turn enhance chemokine and cytokine production through the PI3K-NF-κB signaling axis (126). This pro-inflammatory activity aligns with its expression in macrophages (127), dendritic cells (128), and microglia (129). Elevated EREG levels in the serum and joints of rheumatoid arthritis patients (126, 130) as well as in leukocytes of orofacial pain patients (26) further supports its pathological role in pain. Interestingly, genetic deletion of EREG reduces IL-6 expression in antigen-presenting cells (131), and monoclonal EREG antibody (**Table 2**) relieves acute and chronic pain behaviors in rodents (33). Collectively, these findings suggest that the EREG-EGFR axis is a critical component of pain pathology and may contribute to postoperative pain susceptibility.

**Table 2:** The role of inflammatory mediators in postoperative pain.

Ligand	Receptors	Modulated molecules	Inhibitors	Analgesic effect	References
TNFα	TNFR1 TNF2	↑ Na^+^ channels ↑Na^+^ currents ↑AMPAR	Infliximab PDTC SB203580	↓Mechanical allodynia ↓ Nociceptive activity in somatosensory cortex, thalamus and limbic system	(108, 165, 171)
IL-1β	IL-1R1 IL-1R2	↑ Na^+^ channels ↑AP threshold	rIL-1ra SB203580	↓ Mechanical allodynia ↓Thermal hyperalgesia	(194, 195)
IL-6	mIL-6R sIL-6R gp130	↑TRPV1 ↑TRPA1	Anisomycin gp130^-/-^ elF4E^-/-^	↓Mechanical allodynia ↓AP threshold shift ↓Thermal hyperalgesia	(216, 217, 220)
Chemokines	CCR2 CCR4 CCR8	TRPV1 TTX-R VGSCs AMPARs	INCB3344 anti-CCL1 NASPM CCL7mAb GSK-J4 Akt IV MK2206 Rapa Rida	↓Thermal hyperalgesia ↓Cold allodynia ↓Mechanical allodynia ↓Pain behaviors	(270–272, 274, 278, 282)
PGE_2_	EP1 EP2 EP3 EP4	↑Ca^2+^ currents ↑Na^+^ currents ↓K^+^ currents, ↓Glycine receptor	CJ-023 CJ-042 MF266-1 MF266-3 anti-PGE_2_	↓Edema ↓Thermal hyperalgesia ↓Pain behavior	(346–349)
MMPs	N/A	Cytokines, growth factors and extracellular matrix	444278 444288	↓Thermal ↓Mechanical allodynia	(315, 316)
IL-10	IL-10Rα IL-10Rβ	↓Na^+^ currents ↓Na^+^ channel	rIL-10 ODN IMT504	↓Postsurgical pain ↓Mechanical allodynia ↓Cold allodynia	(239, 240)
TGF-β1	TβRI TβRII	↑opioid receptors ↑opioids	Monnieriside A BAMBI^-/-^	↓Pain behavior ↓Mechanical allodynia	(253, 255, 256)
EGF HB-EGF TGFα AREG EREG	EGFR	↑Glutamate receptors ↑TRPV1 channels	NBP2-21992 AG1478 C225 AMG 9810 Lapatinib Gefitinib Cetuximab	↓ Mechanical allodynia ↓ Nocifensive behaviors	(26, 33, 37, 75, 125, 131, 134)
Hyaluronic acid Fibrinogen HMGB1 Heat shock proteins LPS	TLR4	↑Na^+^ channels ↑Microglia activation	Naltrexone Naloxone Mutant LPS LPS-RS FP-1 TAK-242	↓Mechanical allodynia ↓Thermal hyperalgesia	(142, 143, 148–150)

Similarly AREG may contribute to pain pathology as elevated AREG levels correlate with sepsis severity and activate NF-κB-dependent cytokine production (132, 133). In neuropathic pain models AREG expression correlates with injury induced neuronal hyperexcitability while deletion or knockdown of AREG alleviates pain behaviors (111). In this study, AREG-EGFR signaling induced chemokine and COX-2 expression, reinforcing its contribution to neuroinflammation and pain.

Epidermal growth factor also directly modulates pain signaling. In keratinocytes, EGF induces EGFR phosphorylation and downstream activation of ERK1/2 and c-Jun, enhancing chemokine production, an effect reversed by erlotinib (134). Additionally, intrathecal injection of EGF produces mechanical hypersensitivity in rodents, which is reversed by imatinib and prevented by gefitinib (70).

These findings establish EGFR as a potent regulator of pain at peripheral, and CNS levels. The consistent analgesic effects observed with EGFR inhibitors, often at doses below those required for anticancer efficacy, highlight opportunities for therapeutic repurposing in pain management. However, these effects have not been demonstrated in postoperative pain where opioids are used during surgical procedures.

### 4.2 EGFR and TLR4 signaling in pain

In addition to ligand-dependent activation, EGFR interacts with toll like receptors(TLRs) during immune responses to infection and tissue injury (90–92, 135). Following sterile tissue injury, TLR4 is activated by DAMPs including hyaluronic acid, fibrinogen, heat shock proteins, and HMGB1, initiating innate immune responses (136, 137). DAMP binding causes TLR4 conformational changes that recruit adaptor proteins (**Figure 1**), launching NF-κB, MAPK, AP-1, and IRF5 pathways (138) leading to increased expression of inflammatory mediators (**Figure 1**). Notably, increased TLR4 expression has been reported in multiple pain models including peripheral mononeuropathy (139), CFA-induced inflammation (140, 141) and postoperative pain (142, 143). Cellular TLR4 expression is observed in microglia, astrocytes, and sensory neurons, where it contributes to the development and maintenance of chronic pain (144–147). One way that TLR4 modulates pain is through activation of pathways that promote sodium channels transcription and activate microglia (143). Consequently, inhibition of TLR4 reverses mechanical hypersensitivity across neuropathic, inflammatory, and postoperative pain models (**Table 2**) (142, 148–150). In a rodent model of postoperative pain, TLR4 and NF-κB are upregulated, in the DRG and incised tissue, and TLR4/NF-κB inhibition reduced cytokine expression and attenuated hyperalgesia (142).

Crosstalk between TLR4 and EGFR is well documented, with evidence indicating that EGFR phosphorylates TLR4 to allow binding of effector molecules (151). This is supported by studies showing that pharmacological inhibition of EGFR blocks LPS-induced microglial chemotaxis (75), and suppresses proinflammatory cytokine production in macrophages (37, 75). Consistent with these findings, EGFR deletion reduces LPS-induced expression of TLR4 as well as downstream inflammatory mediators including IL-1β, IL-6, and TNFα (152), indicating that EGFR signaling is required for optimal TLR4 activation. Mechanistically, EGFR has been shown to facilitate TLR4 endosomal trafficking (**Figure 1**) (152), providing a plausible explanation for suppression of TLR4 dependent cytokine production observed in EGFR inhibition. Collectively, these data support a model in which surgical tissue injury releases DAMPs that activate TLR4 to initiate cytokine production, while concomitant EGFR activation promotes TLR4 endosomal trafficking amplifying the inflammatory response (**Figure 1**).

Despite strong evidence for EGFR-TLR4 crosstalk, this signaling axis has not been investigated in the context of postoperative pain. Given the central role of both EGFR and TLR4 in inflammatory signaling, targeting this pathway may represent a promising therapeutic strategy to attenuate surgery-induced pain and inflammation.

### 4.3 The PI3K/Akt/mTOR signaling pathway in postoperative pain

The PI3K/Akt/mTOR pathway is activated during peripheral nerve injury (PNI) (24) and its inhibition suppresses pain behaviors in rodent models (26, 153–156). This pathway promotes ion channel expression (153) and neuroinflammation (157), processes that contribute to acute to chronic pain transition.

A proteomic screen for pronociceptive factors released by cancer cells identified EGFR as the major driver of their pain-promoting activity in rodents (125). Cetuximab, an EGFR antibody, (**Table 2**) abolished the pronociceptive properties of cancer supernatants, and downstream pathway analysis revealed the activation of PI3K-Akt-mTOR signaling in recipient mice (125). Additional evidence comes from a bone cancer pain model, where PI3K-mTOR inhibition not only alleviated pain but also improved locomotion and affective behaviors (158).

**Figure 2:**
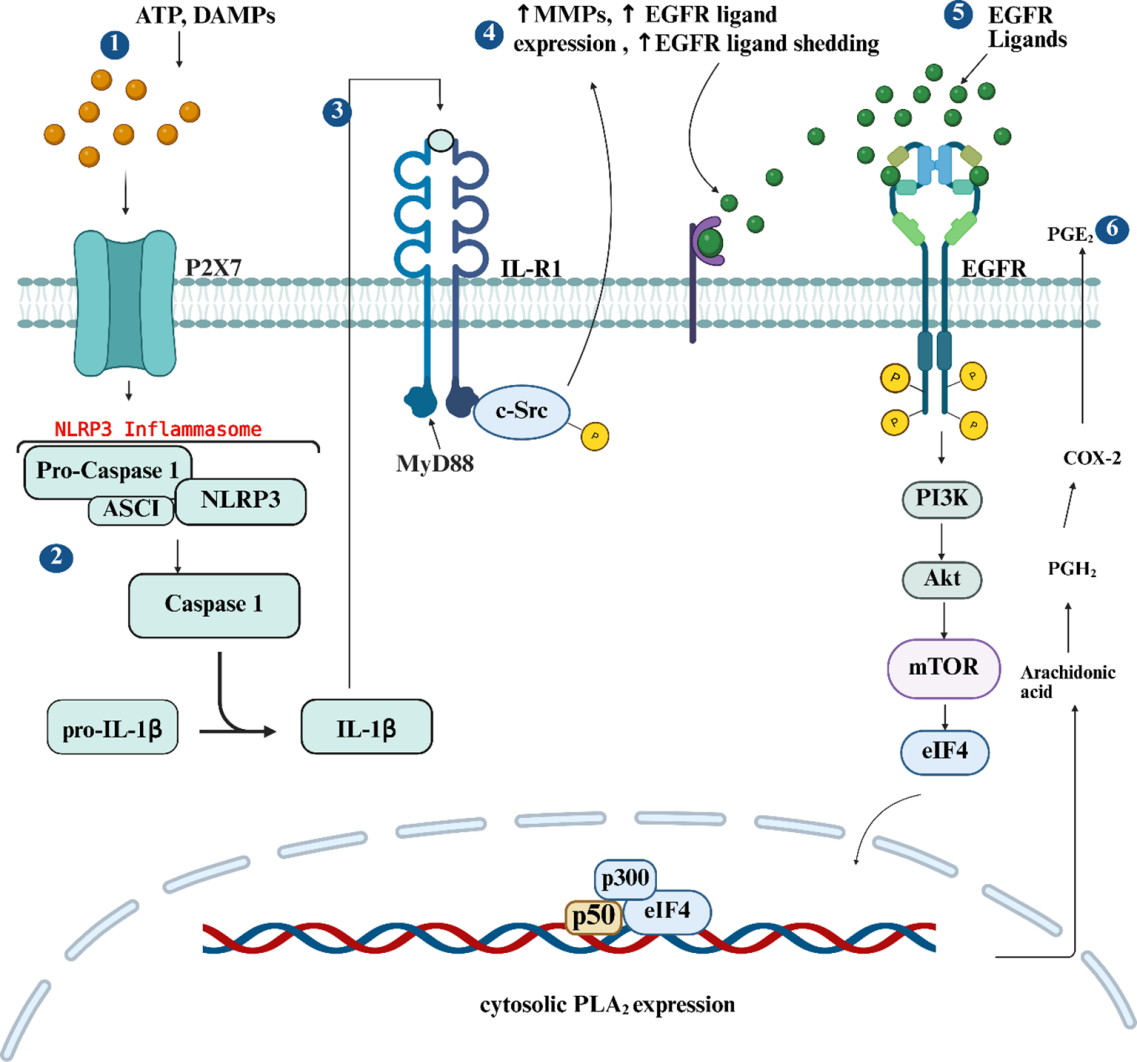
EGFR and IL-1β signaling mechanisms in peri-incision tissue as well as synovial fibroblasts and epithelial cells. (1) Sterile surgery releases ATP which acts through its receptor to activate the (2) NLPR3 inflammasome and cleavage of pro-1β. (3) IL-1β then binds to its receptor to upregulate MMPs and EGFR ligand expression. (4) Pro-EGFR ligands are then cleaved by MMPs, and (5) bind to EGFR which activates the PKI3-Akt-mTOR leading to transcription of inflammatory mediators. (6) The EGFR-PKI3-Akt-mTOR also promotes transcription of cytosolic LPA_2_ which initiates the conversion of arachidonic acid to PGE_2_. Created in https://BioRender.com.

Surgery activates Akt/mTOR signaling (in spinal and peripheral neurons), and pharmacological inhibition of either protein reduces incision-induced neuronal activation and hypersensitivity (23). Similarly, post-surgical PI3K inhibition suppresses pain behavior through reduced Akt signaling in neurons and microglia (159), and PI3K/Akt/mTOR inhibitors attenuate pain behavior in chronic postoperative pain (27). Downstream of mTOR is the eukaryotic translation initiation factor 4E (eIF4E) which controls neuronal protein synthesis and synaptic plasticity (160) (**Figure 2**) demonstrating that activation of this pathway mediates acute pain to chronic pain transition. In addition to EGFR, this pathway can be activated by other receptors including mGluR1and Trk (160), but there is little information that connects EGFR signaling to this pathway in postoperative pain.

### 4.4 EGFR and cytokines

Inflammation is orchestrated through complex signaling networks in which cytokines and growth factor receptors function in a highly interconnected manner. Among these, EGFR has emerged as a critical signaling node that integrates inflammatory cues with cellular responses traditionally associated with growth, repair, and survival. Beyond its canonical activation, EGFR is frequently activated through cytokine-driven transactivation mechanisms, enabling inflammatory mediators to amplify downstream signaling cascades. This bidirectional crosstalk allows EGFR to modulate key inflammatory pathways, thereby influencing cytokine production, immune cell recruitment, and tissue remodeling. Understanding the mechanisms and consequences of these interactions is essential for elucidating their contribution to tissue injury induced inflammation and pain.

#### 4.4.1 Tumor necrosis factor

Tumor necrosis factor alpha is a pleiotropic cytokine secreted by macrophages, natural killer cells, astrocytes, microglia, Langerhans cells, Kupffer cells and alveolar macrophages (161, 162). TNFα signals through two receptors, tumor necrosis factor receptor 1(TNFR1) and tumor necrosis factor receptor 2 (TNFR2) to activate intracellular mechanisms that promote tissue regeneration, proliferation, differentiation, survival and apoptosis (163). Both receptors are expressed on sensory neurons including afferent nociceptive neurons (164).

**Figure 3:**
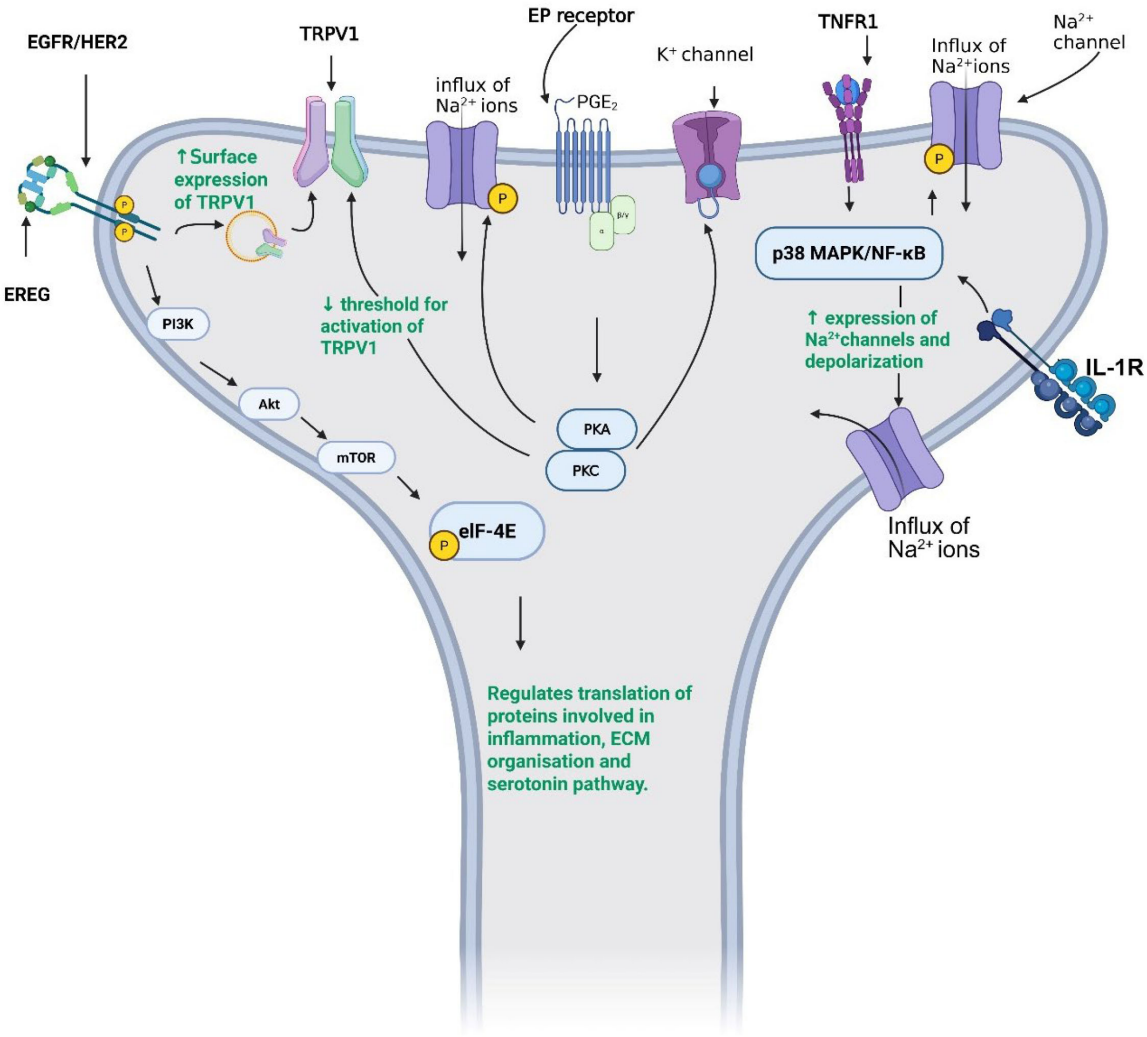
Peripheral mechanisms that lead to nociceptor activation following surgery. Nociceptors, including Aδ- and C-fibers, express cytokine, EGFR, and EP receptors. EGFR signaling in nociceptors facilitates TRPV1 endosomal recycling, enhances translation of extracellular matrix (ECM)-related proteins, and activates serotonin-related pathways, each contributing to pain facilitation. Activation of EP receptors leads to phosphorylation of Na^+^ channels, increasing Na^+^ influx, while simultaneously reducing K^+^ channel activity and preventing hyperpolarization. Cytokines further sensitize nociceptors by increasing Na^+^ channel expression and Na^+^ influx. Created in https://BioRender.com.

Using the plantar incision model, de lima et al. (108), show that TNFα expression is upregulated in the skin and DRG of injured mice and that this upregulation drives post-surgery mechanical hypersensitivity. TNFα acting through its receptor, TNFR1, initiates a signaling cascade that culminates into the activation of p38 MAPK (164) which upregulates and phosphorylates voltage gated sodium channels (VGSCs) to increase influx of sodium ions into afferent nociceptive neurons (**Figure 3**) (165). Notably, pharmacological antagonism of p38 MAPK downregulates mRNA for *Na_v_1.8* and *Na_v_1.9* sodium channels reducing mechanical hypersensitivity (108). Furthermore, TNFα increases surface expression of a-amino-3-hydroxyl-5-methyl-4-isoxazole-propionate (AMPA)/kainate (Ca-A/K) receptor which enhances influx of calcium ions, a mechanism proposed to facilitate acute to chronic pain transition (166–169). TNFα has also been implicated in the pathogenesis of perioperative neurodegenerative disorders since increased expression of this cytokine in the medial pre-frontal cortex (mPFC) and hippocampus, of rats undergoing internal fixation of the tibia fracture, was associated with the development of spatial memory impairment (170). Interestingly, blockade of TNFα with infliximab (**Table 2**) blocks nociceptive activity in the somatosensory cortex and thalamus as well as the limbic system (171).

TNFR and EGFR signaling exhibit bidirectional and context-dependent crosstalk that critically regulates cell fate during inflammation. These responses are shaped by TNFα and its downstream effector pathways to generate tissue-specific outcomes. Current evidence indicates that TNFα signaling through TNFR mediates phosphorylation and transactivation of EGFR via downstream MAPK/NF-κB (45, 172, 173), PI3K/Akt (174), and JNK/FoxO1 (175) signaling cascades. These studies consistently show that TNFα increased EGFR receptor expression and phosphorylation exacerbating tissue damage. Importantly, TNFR-EGFR interactions are also evident in the CNS, where TNF-α induces sympathetic excitation in cardiovascular regions of the forebrain via EGFR and ERK1/2 signaling (54) increasing blood pressure and heart rate in rats. Conversely, EGFR inhibition promotes TNFR-mediated apoptosis (176–178). In fact, loss of EGFR ligand shedding in the presence of sustained TNFR1 activity skews TNFR signaling toward apoptosis (179) and pharmacologic or genetic blockade of EGFR sensitizes cells to TNF-induced apoptosis (180). These studies demonstrate that EGFR-TNFR crosstalk functions as a molecular rheostat, balancing survival and apoptotic signaling during inflammation. The ultimate cellular outcome is dictated by cell type and downstream signaling bias, favoring either pro-survival pathways (178) or pro-death and inflammatory pathways (176, 177).

#### 4.4.2 Interleukin-1β

A key step in IL-1β maturation is activation of the NLRP3 inflammasome, which assembles in response to cellular danger signals such as extracellular ATP (**Figure 2**) (181), matrix degradation products (182, 183) and reactive oxygen species generated during tissue injury (184). Surgical incision upregulates NLRP3, leading to increased IL-1β activation, while genetic deletion of NLRP3 reduces both mechanical and thermal hypersensitivity in the plantar incision model (185). At the peripheral level, incision injury elevates IL-1β expression in the incised skin and DRG (186–187), where IL-1β can directly excite nociceptors (188–191). Intrathecal administration of IL-1β reliably induces hyperalgesia and allodynia (192, 193), while pharmacological blockade of IL-1R1 (**Table 2**) attenuates postoperative hypersensitivity (194). Electrophysiological studies show that IL-1β activates p38 MAPK, enhancing sodium currents and shifting channel activation toward more depolarized potentials (**Figure 3**). These changes increase firing frequency and lower activation thresholds, effectively sensitizing nociceptors (195). Notably, the p38 MAPK inhibitor SB203580 (**Table 2**) prevents these IL-1β-induced effects, underscoring the importance of IL-1R/MAPK in postoperative nociception.

While IL-1β classically signals via the IL-1receptor (IL-1R), a substantial body of evidence demonstrates that IL-1β also engages and amplifies EGFR-dependent signaling through both ligand-dependent and ligand-independent mechanisms. IL-1β induces cytosolic phospholipase A_2_ (cPLA_2_) expression and subsequent PGE_2_ release via MMP-induced HB-EGF shedding, a process involving epigenetic control of inflammatory gene expression (**Figure 2**) (196). Synergistic interactions between IL-1β and EGF further potentiate inflammatory outputs via transcriptional regulation of inducible nitric oxide synthase(iNOS) (197). Consistent with this cooperative paradigm, studies in human thymic epithelial cells reveal that EGF and TGF-α regulate IL-1α, IL-1β, and IL-6 expression predominantly at the post-transcriptional level (198).

IL-1β interactions with EGFR promote ligand expression, proteolytic release, and receptor trafficking. In the context of rheumatoid arthritis (RA), IL-1β selectively upregulates AREG at both the mRNA and protein levels in human fibroblast-like synovial cells (FLS-RA), engaging intracellular cascades such as p38 MAPK, NF-κB, JNK, and ERK1/2 that drive AREG synthesis, as well as PI3K, p38, and NF-κB pathways that induce MMP expression (**Figure 2**) (199). The resulting AREG then enhances MMP-1 and cadherin-11 expression to promote FLS invasive behavior characteristic of arthritic joints. IL-1β-driven EGFR ligand regulation is also evident in compromised cystic fibrosis transmembrane conductance regulator (CFTR) function which increases IL-1β secretion, establishing an autocrine IL-1β loop that elevates EREG expression (46) amplifying inflammation. Beyond ligand induction, IL-1β directly modulates EGFR receptor dynamics via activation of the p38 MAPK and downstream effector proteins phosphorylating EGFR which causes receptor internalization (200). This internalization correlates with altered cell morphological changes characteristic of loss of cell-cell contact, suggesting IL-1β can promote features associated with epithelial-mesenchymal transition (EMT) via receptor EGFR endosomal trafficking.

Collectively, these studies illustrate a multifaceted role for IL-1β in modulating EGFR signaling network: (1) upregulating EGFR ligands such as AREG and EREG through direct cytokine receptor signaling, (2) facilitating proteolytic release of these ligands via MMPs to promote invasive and inflammatory phenotypes, and (3) altering receptor phosphorylation and trafficking to modulate cellular responses independent of direct ligand binding. This integrated IL-1β-EGFR interplay contributes to tissue-specific inflammatory pathologies, from invasive synovitis in RA to epithelial dysfunction and remodeling in intestinal and pulmonary tissues.

#### 4.4.3 Interleukin-6

Interleukin-6 (IL-6) is a pleiotropic cytokine that regulates immune responses, tissue repair, metabolism, pain and neural plasticity primarily through activation of the JAK/STAT3 pathway. Interleukin-6 (IL-6) is consistently elevated following surgical injury and is one of the most reliable inflammatory markers linked to postoperative pain and recovery outcomes. In rodent incision models, IL-6 expression increases rapidly in injured tissue (201), and exogenous IL-6 produces robust mechanical and thermal hypersensitivity (202, 203). Blocking IL-6 activity, either genetically or through neutralizing strategies, attenuates these responses and, in some cases, prevents the development of chronic hypersensitivity (**Table 2**) (204–206). Similar trends have been documented in clinical settings where higher circulating IL-6 levels correlate with increased postoperative pain intensity, delayed recovery, and a greater likelihood of persistent postoperative pain (207–214). Genetic deletion or knockdown of IL-6R subunit, gp130, diminishes IL-6 evoked hypersensitivity (203, 215–217).

IL-6 signaling increases the expression of TRPV1 and TRPA1 channels, thereby lowering the thresholds for channel activation (215, 218). IL-6 also engages translational control pathways via ERK-dependent phosphorylation of eIF4E. In mice lacking eIF4E phosphorylation (**Table 2**), IL-6 fails to produce mechanical or thermal hypersensitivity (219), and treatment with anisomycin (**Table 2**) attenuates IL-6-induced allodynia (220).

Substantial evidence demonstrates that IL-6 signaling is closely integrated with EGFR pathways, forming a bidirectional signaling network that amplifies immune responses and pathological conditions. Across diverse cell types, IL-6 and EGFR signaling converge through JAK/STAT3 (221), MAPK/ERK (48, 221–223), and NF-κB (224) where EGFR ligands increase both IL-16 mRNA and protein expression. Conversely, IL-6 induces secretion EGF ligands (225), via metalloproteinase-mediated shedding of EGFR ligands (226). This coordinated regulation activates an autocrine feedback loop that amplifies inflammation with subsequent tissue remodeling and chronic inflammation. Consistent with this paradigm, experiments in rodent models of arthritis reveal that IL-6 signaling activates EGFR and that inhibition of EGFR attenuates IL-6-induced neuronal sensitization (84), affirming that EGFR and IL-6 crosstalk contributes to neuronal hyperexcitability.

#### 4.4.4 Interleukin-10

IL-10 is a key anti-inflammatory cytokine that counterbalances the proinflammatory responses triggered by surgical injury. Originally identified for its ability to suppress Th1 cytokine production (227), IL-10 is now understood to regulate a broad spectrum of immune cells, including macrophages, dendritic cells, T cells, natural killer cells, and B cells (228–231). Within the CNS, IL-10 is produced primarily by microglia and astrocytes, although infiltrating immune cells become a dominant source during injury or inflammation (232). IL-10 signals through a heterotetrameric receptor composed of IL-10Rα and IL-10Rβ, initiating downstream pathways that suppress proinflammatory cytokine production and limit collateral tissue damage (231, 233). A substantial body of evidence demonstrates the analgesic potential of IL-10 (234–242). For instance, pretreatment with IL-10 attenuates dynorphin-evoked hypersensitivity (235), and intrathecal delivery of recombinant IL-10 or IL-10 encoding viral vectors transiently reverses mechanical and thermal hypersensitivity following nerve injury (**Table 1**) (234). Although the short half-life of rIL-10 and rapid immune clearance of viral vectors limited IL-10 efficacy, these studies establish IL-10 as a potent endogenous modulator of pain. In DRG cultures, IL-10 reduces sodium currents by decreasing sodium channel expression (239), thereby lowering neuronal excitability. Therefore, strategies that increase endogenous IL-10 levels, such as immunomodulatory oligodeoxynucleotides (**Table 2**) (240), significantly diminish postsurgical pain behaviors. Taken together, IL-10 serves as a critical brake on postoperative inflammatory signaling, acting both on immune cells and nociceptors to limit the development of central and peripheral sensitization.

EGFR is as a critical regulator of immune homeostasis, functioning beyond its classical roles in epithelial proliferation and tissue repair. In immune and barrier tissues, EGFR signaling modulates cytokine production, macrophage polarization, and resolve inflammation via elevation of IL-10. Evidence from mechanistic studies and inflammatory disease models supports a functional EGFR-IL-10 axis that is highly context dependent and essential for balanced immune responses. Several experimental studies demonstrate that EGFR regulates macrophage cytokine production via STAT3 and PI3K/AKT, signaling pathways that drive IL-10 transcription.

Direct evidence linking EGFR to IL-10 production comes from inflammatory disease models using macrophage-specific EGFR manipulation. In dextran sulfate sodium (DSS)-induced colitis, mice lacking EGFR, specifically in macrophages, exhibited significantly increased IL-10 expression in colonic tissue compared with wild-type controls (41). In this study, EGFR knockout increased IL-10 mRNA and protein levels during both the acute inflammatory phase and the recovery phase. This effect correlated with reduced colonic damage, cryptic damage and inflammation. Importantly, neutralization of IL-10 reversed this protective phenotype, establishing IL-10 as a necessary mediator of inflammatory resolution in this EGFR-dependent context. Complementary *in vitro* studies using bone marrow-derived macrophages further support the role for EGFR signaling in IL-10 upregulation (105) where EGFR inhibition reduced the M1 phenotype. However, genetic deletion of EGFR reduced IL-10 expression in gastric tissues and increased bacteria burden, this effect was opposite in macrophages where EGFR inhibition/knockout increased the production of IL-10 (105), indicating that EGFR regulation of IL-10 expression is tissue specific. Similarly in wound healing studies EGFR activation elevates IL-10 however, EGFR inhibition prevents keratinocyte proliferation and migration which slows down wound closer (243).

A consistent mechanistic theme across preclinical models is the involvement of the EGFR-STAT3 signaling axis in promoting anti-inflammatory immune phenotypes. EGFR-mediated STAT3 phosphorylation has been shown to drive macrophage and microglial polarization towards M2-phenotype; characterized by increased IL-10 production and suppression of pro-inflammatory cytokines (95). In models of neuroinflammation and CNS injury, EGFR-dependent STAT3 activation promotes microglial M2 polarization and attenuates inflammatory damage (95, 244). While EGFR inhibition may reduce acute inflammatory signaling, excessive or prolonged inhibition may inadvertently disrupt IL-10-mediated immune regulation, predisposing tissues to chronic inflammation or delayed repair (245). Thus, understanding the context-specific role of EGFR in IL-10 regulation is critical for optimizing EGFR-targeted interventions.

In summary, converging evidence from inflammatory disease and immune cell models supports a functional EGFR-IL-10 axis in macrophages and barrier tissues, influencing cytokine production and tissue repair. Together, these findings demonstrate that EGFR activation upregulates IL-10 production, with effects that depend on inflammatory timing, tissue microenvironment, and macrophage activation state.

#### 4.4.5 Transforming growth factor

Transforming growth factor-β (TGF-β) is a multifunctional cytokine family composed of three mammalian isoforms, TGF-β1, TGF-β2, and TGF-β3, that regulate immune homeostasis, tissue remodeling, and neuronal function. Genetic deletion studies highlight the essential nature of these molecules: loss of TGF-β2 or TGF-β3 disrupts development, while TGF-β1 deficiency produces severe multisystem inflammation leading to early lethality (246–248). Of these isoforms, TGF-β1 is most strongly associated with modulation of inflammation and pain. TGF-β signals through a receptor complex formed by TβRI and TβRII serine/threonine kinases (249), which are widely expressed across CNS regions including the cortex, brainstem, spinal cord, midbrain, and hippocampus (250–252). Under normal conditions, the pseudoreceptor, bone morphogenetic protein and activin membrane-bound inhibitor (BAMBI) restrains TGF-β signaling, and its deletion enhances TGF-β1 activity and reduces pain-related behaviors in inflammatory and neuropathic models (**Table 2**) (253). Exogenous TGF-β1 administered intrathecally similarly suppresses mechanical and thermal hypersensitivity after nerve injury (254). The analgesic properties of TGF-β1 appear to arise from its ability to upregulate μ- and δ-opioid receptors and increase production of endogenous opioid peptides such as β-endorphin and enkephalin (253, 255, 256). This modulatory effect extends to postoperative pain where increasing TGF-β1 expression reduces incision-induced hypersensitivity (256), suggesting that boosting endogenous TGF-β1 signaling may counteract early inflammatory cascades and provide durable analgesia.

Transforming growth factor-β (TGF-β) and EGFR signaling pathways are central regulators of tissue homeostasis, repair, and pathological remodeling. Although traditionally viewed as distinct signaling systems, extensive evidence demonstrates bidirectional and context-dependent crosstalk between TGF-β and EGFR that shapes cellular responses in vascular, epithelial, and mesenchymal compartments.

Early mechanistic studies demonstrate that TGF-β can induce rapid EGFR phosphorylation through ligand-independent mechanisms involving intracellular kinases and redox signaling (257, 258). Subsequent work identified metalloproteinase-dependent shedding of EGFR ligands as another mechanism that TGF-β utilizes to activate EGFR (259). In hepatocytes, TGF-β activates NADPH oxidase (NOX1), leading to NF-κB-dependent upregulation of EGFR ligands and downstream receptor activation (260, 261). This EGFR-TGF-β crosstalk has functional consequences for ECM production and tissue remodeling. In vascular biology, loss of the ECM protein, EMILIN-1, enhances arteriolar myogenic tone through TGF-β-dependent EGFR transactivation, contributing to hypertensive phenotypes in both murine models and humans (262). In airway and epithelial repair models, wound-induced TGF-β1 and TGF-β2 stimulate epithelial regeneration via induction of EGFR ligands to facilitate repair responses (263). Similarly, EGFR-driven TGF-β production in airway epithelium and cardiac fibroblasts establishes a feed-forward loop characterized by proteolysis (259).

In fibroblasts, EGFR antagonizes TGF-β-induced tropoelastin (via stabilization of TGF-β transcriptional repressor) leading to decline in elastin deposition and subsequent failure of tissue repair processes (264, 265). In contrast, EGFR activation is required for TGF-β-mediated fibronectin expression in chondrocyte progenitor cells and cardiac fibroblasts (266, 267). Furthermore, EGFR activation of TGF-β facilitates collagen synthesis and vascular remodeling leading to increased systolic blood pressure (268). These findings demonstrate that EGFR can either potentiate or restrain TGF-β responses depending on cellular context.

Collectively, these studies establish EGFR as a critical regulator of TGF-β signaling. Through ligand-dependent and ligand-independent mechanisms, EGFR shapes TGF-β-driven outcomes in fibrosis, vascular tone regulation, epithelial repair, and inflammatory remodeling. Dysregulation of this crosstalk contributes to pathological states including hypertension and airway remodeling.

### 4.5 EGFR and chemokines

Chemokines form a large family of low-molecular-weight signaling molecules that participate in leukocyte recruitment, tissue repair, and the coordination of innate and adaptive immunity. Increasing evidence suggests that they also directly modulate nociceptive processing. Surgical injury induces rapid upregulation of chemokines both in injured tissue (8, 269, 270) and within the nervous system (271–275), and many sensory neurons express chemokine receptors capable of responding to these chemokines (276). Several chemokines have emerged as key contributors to postoperative hypersensitivity. C-C motif chemokine ligand 2(CCL2), for example, increases the expression of TRPV1 and Na*_v_*1.8 channels in DRG neurons (277), enhances excitability, and produces mechanical and thermal hypersensitivity when administered intrathecally (**Table 2**) (278). Antagonism of its receptor, CCR2, blocks peripheral transport of neuropeptides and significantly alleviates pain behavior in rats (278, 279). Similar roles have been described for CCL1 and CCL7 where neutralizing antibodies targeting each chemokine attenuate mechanical and cold hypersensitivity in tibia fracture and incision models (**Table 2**) (271, 272). Skin-resident dendritic cells also contribute to postoperative pain through their release of the CCR4 ligands, CCL17 and CCL22. These chemokines evoke rapid thermal and mechanical hypersensitivity that can be reversed by CCR4 knockdown (270). Downstream signaling involves activation of ERK, Akt, JNK, and mTOR pathways (23, 159, 275, 280–282), which regulates ion channel expression, synaptic plasticity, and long-term pain facilitation. Persistent JNK activation, for instance, promotes chronic pain by modifying ion channel function (274, 283) and enhancing expression of neuropeptides and matrix metalloproteinases (284–286). These findings illustrate that chemokines are key modulators of acute pain, raising the possibility that therapeutic targeting might offer early pain relief preventing the transition of acute to chronic pain.

A conserved feature of EGFR-chemokine interaction is metalloproteinase-dependent shedding of EGFR ligands, including AREG, HB-EGF, and TGF-α. Proinflammatory stimuli such as TNF-α, macrophage inflammatory protein-3α (CCL20) (175), neurotensin (287), protease-activated receptor-2 (PAR2) agonists (288), and leukotrienes (289) activate ADAM metalloproteinases, resulting in rapid EGFR ligand release and receptor phosphorylation. This mechanism has been consistently demonstrated in airway (290–293), intestinal (294–296), and cardiac cells (175, 287) (**Table 3**), where EGFR transactivation is required for chemokine gene transcription. Metalloprotease-dependent ligand shedding, src kinase and GPCR signaling are key pathways that mediate EGFR-chemokine interaction (287, 293). For example, inflammatory mediators such as lysophosphatidic acid (LPA) (297, 298), as well as thrombin (299) and bradykinin (290), activate EGFR causing increased chemokine expression.

**Table 3:** EGFR-chemokine interactions in inflammation.

Stimulus	Cell type	Mode of EGFR transactivation	Key findings	Reference(s)
TNF-α	Airway epithelial cells	Metalloproteinase-mediated AREG shedding	↑CXCL8 (IL-8)	(292)
Neutrophil Elastase	Lung epithelial cell line	Metalloproteinase-mediated EGFR ligand shedding	↑CXCL8	(300)
Leukotriene D_4_ (LTD_4_)	Bronchial epithelial cells	Metalloproteinase-mediated HB-EGF shedding	↑CXCL8	(298)
Lysophosphatidic Acid (LPA)	Bronchial epithelial cells	Not determined	↑CXCL8	(297, 298)
Thrombin	Human osteoblasts	src-mediated EGFR phosphorylation	↑CCL2 (MCP-1)	(299)
TNF-α	Human cardiac fibroblasts	Not determined	↑CCL20	(175)
TNF-α	Normal human epidermal keratinocytes	Metalloproteinase-mediate AREG shedding	↑CCL27	(302)
Neurotensin	Human colonic epithelial cells	Metalloproteinase-mediated TGF-α shedding	↑CXCL8	(287)
Ozone	Human bronchial epithelial cells	src-mediated EGFR phosphorylation	↑CXCL8	(293)
Hypoxia	Fibrocytes	Not determined	↑CXCR4	(304)
*Streptococcus suis* serotype 2 (SS2), strain SC19	Human brain microvascular endothelial cells (hBMEC)	Metalloproteinase-mediated HB-EGF, AREG and EREG shedding	↑bacterial-induced neuroinflammation (↑IL-8, ↑MCP-1, ↑MIP-2)	(306)
*Clostridium difficile* Toxin B	Non-transformed human colonic epithelial cells	Metalloproteinase-mediated TGFα shedding	↑CXCL8	(295)

CXCL8 (interleukin-8) represents the most extensively characterized chemokine regulated by EGFR transactivation. Numerous studies demonstrate that diverse inflammatory stimuli, including neutrophil elastase, ozone, LPA, LTD_4_, thrombin, and PAR2 agonists (289, 293, 297, 299, 300), require EGFR activation for CXCL8 gene transcription and protein release (**Table 3**). Downstream signaling commonly involves p38 MAPK, ERK1/2, and NF-κB, which together promote transcriptional activation and mRNA stabilization of CXCL8 (288, 292, 300). Importantly, EGFR inhibition or blockade of ligand shedding attenuates CXCL8 production, even when the initiating stimulus does not directly engage EGFR (287–289, 293). These findings establish EGFR as a key signaling intermediate for maximal CXCL8 expression and suggest that EGFR is a key checkpoint in neutrophil-driven inflammation. Besides CXCL8, EGFR transactivation modulates CC chemokine signaling pathways in osteoblasts (299), vascular smooth muscle cells (301), and cardiac fibrocytes (175). However, EGFR signaling can also suppress chemokine expression (302), indicating that the outcome of EGFR and chemokine interaction depends on cellular and disease context.

EGFR-chemokine crosstalk has been implicated in the pathogenesis of chronic inflammatory diseases, including asthma (303), chronic obstructive pulmonary disease (COPD) (304, 305), inflammatory bowel disease (287), cardiovascular inflammation (175), and neuroinflammatory (306). In COPD, hypoxia-induced EGFR/HIF-1α signaling promotes fibrocyte activation (304), causing tissue remodeling. In infectious settings, such as *Helicobacter pylori* infection and *Streptococcus suis* meningitis (296, 306), EGFR transactivation contributes to epithelial and neuroinflammation, respectively (**Table 3**). Collectively, these studies identify EGFR as a signaling nexus that integrates chemokine cues to coordinate immune cell recruitment, and tissue responses. Given its central role in amplifying chemokine-driven inflammation, EGFR represents an attractive therapeutic target especially in postoperative inflammation where infiltration of immune cells worsens tissue injury and pain.

### 4.6 EGFR, metalloproteinases and prostaglandins

#### 4.6.1 Matrix metalloproteinases

Matrix metalloproteinases (MMPs) are zinc-dependent proteases that regulate extracellular matrix remodeling (307, 308) and modulate cytokine activity. Although essential for normal tissue repair, dysregulated MMP activity contributes to both inflammation and pain (309). Among the MMP family, MMP-2 and MMP-9 have been consistently linked to nociceptive sensitization (310–313). Peripheral nerve injury and inflammatory stimuli increase expression of both enzymes in DRG neurons, astrocytes, and microglia (310, 313, 314), and intrathecal administration of MMP-2 or MMP-9 produces mechanical hypersensitivity in rodents (310). Postoperative pain models highlight a particularly important role for MMP-9. MMP-9 knockout mice fail to develop mechanical hypersensitivity following plantar incision, and wild-type animals exhibit increased spinal MMP-9 expression and activity after surgery (315). Inhibiting MMP-2/9 reduces postoperative pain behaviors and simultaneously lowers mature IL-1β levels (**Table 2**), indicating that MMP-mediated cleavage of pro-IL-1β contributes to pain sensitization (316).

A recurring theme across literature is that EGFR transactivation by GPCRs and cytokine receptors launches the EGFR-MMP signaling axis. These transactivation events frequently involve MMP-mediated shedding of EGFR ligands (**Table 4**) and subsequent activation of downstream MAPK, PI3K/AKT, JAK/STAT, and src-kinase pathways.Multiple studies demonstrate that EGFR activation leads to increased expression of MMPs (317, 318). In addition, several studies emphasize extensive crosstalk between GPCRs and EGFR that involved increased expression of MMPs (319, 320), including bradykinin (321), cholinergic (322), opioid (323), calcium-sensing (324), and hydroxy-carboxylic acid (325) receptors (**Table 4**). Activation of these receptors activates downstream signaling pathways that increase MMP-expression. This signaling convergence is particularly evident in immune cells, keratinocytes, and fibroblasts, where EGFR amplifies inflammatory gene expression, MMP secretion, and cellular responses.

**Table 4:** Inhibition of EGFR/MMP signaling.

Study	Experimental model	MMP/EGFR antagonism	Key findings	References
EGFR-induced Cell Migration	Esophageal Keratinocytes	MMP-1 and EGFR	↓Keratinocyte Migration	(326)
Articular cartilage degradation	Rat osteochondral explants	EGFR ligand, TGFα	↓Type II collagen and aggrecan degradation	(332)
MOR-EGFR crosstalk	Rat Cortical Astrocytes	MMPs/ADAMs and EGFR	↓Astrocyte Activation	(323)
Schwann Cell Remodeling	Sciatic Nerve Axotomy	MMP-9 and EGFR	↓Schwan Cell Mitosis	(327)
δOR-EGFR transactivation	Rat Cerebral Ischemia Model	MMPs/ADAMs and EGFR	↑Cerebral Ischemic Injury	(60)
AREG-induced MMP-13	Human synovial fibroblasts	MMP-13, AREG and EGFR	↓Cartilage Destruction	(331)
UVB-induced inflammation	Skin Keratinocytes	MMP-1 and EGFR	↓Skin Inflammation	(328)
Notch1-ADAM8 feedback loop	Rat Chondrocytes	ADAM8, MMP9 and EGFR	↓Osteoarthritis Phenotype	(333)
EGFR-TrkA-FPR crosstalk	Human monocytes	MMP9 and EGFR	↓Immune cell activation	(320)
MEGF9 in Osteoarthritis	Cartilage cells	MMP-13 and EGFR	↓Cartilage degradation	(335)
Bradykinin B1 receptor signaling	Human keratinocytes	MMP-2, MMP-9 and EGFR	↓Wound Healing	(321)
AREG and phagocytosis-induced death	Monocytes	EGFR	↑Monocyte apoptosis	(318)
UVB-induced COX-2 expression	HaCaT keratinocytes	MMP-2	↓Skin inflammation	(319)
EGFR inhibition in arthritis	Collagen-induced arthritis (animal)	MMP-3	↓Inflammatory arthritis	(336)
Calcium sensing receptor (CaSR)-EGFR crosstalk	Rat-1 fibroblasts	MMPs and EFGR	↓Fibroblast activation	(324)
Resveratrol protection	Brain endothelial cells	MMP-9	↓Tight junction disruption protein	(330)

In keratinocyte models, EGFR-induced cell migration involves MMP-1 upregulation mediated through the JAK-STAT and MAPK pathways (326). In neural and glial cells, EGFR transactivation by opioid receptor signaling modulates astrocyte and Schwann cell growth (**Table 4**) (323, 327) where MMP-9 mediates EGFR-dependent tissue remodeling and inflammation. In Schwann cells, MMP-9 controls proliferation and phenotypic remodeling via EGFR receptor-dependent activation of MEK/ERK signaling (327). Similarly, in inflammatory and brain injury, MMP-9 inhibition correlates with preservation of tissue integrity and reduced expression of inflammatory markers (319, 328–330). In contrast, increased expression of MMP-9 facilitates wound closer (321) and improves neurological deficits following ischemic injury (**Table 4**) (60) demonstrating that MMP9 inhibition is not always protective but depends on tissue injury context.

Studies focused on degenerative joint diseases, particularly osteoarthritis (OA) and rheumatoid arthritis (RA) show that EGFR ligands such as TGF-α and AREG promote cartilage degradation by inducing MMP expression in synovial fibroblasts and chondrocytes (**Table 4**) (331, 332). In these cell models, EGFR signaling intersects with Rho/ROCK, MEK/ERK, PI3K/AKT/mTOR, and Notch pathways to drive ECM breakdown and disease progression (**Table 4**) (199, 332–335). Consequently, pharmacological inhibition of EGFR or ligand shedding significantly reduced MMP synthesis and cartilage damage in experimental models (331, 332, 336), underscoring the role of EGFR-MMPs axis in chronic inflammatory diseases.

Collectively, the literature indicates that EGFR activation, either through direct ligand binding or transactivation by heterologous receptors, induces MMP activity, which in turn promotes the shedding and availability of EGFR ligands. This establishes a self-reinforcing feed-forward loop that sustains EGFR signaling and contributes to chronic inflammatory responses.

#### 4.6.2 Prostaglandin E_2_

Prostaglandin E_2_ (PGE_2_) is the most abundant and best-characterized prostaglandin involved in pain signaling. It is synthesized from prostaglandin H_2_ (PGH_2_) by three distinct prostaglandin E synthases: microsomal prostaglandin E synthase-1 (mPGES-1), microsomal prostaglandin E synthase-2 (mPGES-2), and cytosolic prostaglandin E synthase (cPGES). Cytosolic PGES is constitutively expressed and functionally couples to cyclooxygenase-1 (COX-1), thereby maintaining basal PGE_2_ levels under homeostatic conditions (337). In contrast, mPGES-1 is inducible in peripheral tissues and preferentially couples to COX-2, driving excessive PGE_2_ production during inflammation (338). Within the central nervous system (CNS), COX-2 is constitutively expressed in the cortex, hippocampus, and amygdala (339, 340), as well as in spinal microglia and projection neurons (341) where it contributes to pain sensitivity. Under physiological conditions, PGE_2_ plays essential roles in regulating immune responses, blood pressure, electrolyte balance, gastrointestinal integrity, and fertility (342). However, pathological overproduction of PGE_2_ is a key driver of inflammation and pain. Consistent with this, intrathecal administration of PGE_2_ induces robust hyperalgesia and allodynia in both mice and rats (343–345). A direct causal role for PGE_2_ in inflammatory pain was demonstrated by Portanova and colleagues, who showed that a neutralizing anti-PGE_2_ monoclonal antibody reverses edema and hyperalgesia (**Table 2**) (346).

PGE_2_ exerts its effects through four E-type prostanoid receptors, EP1-EP4, which are expressed in microglia, astrocytes, and neurons (347–350). Pharmacological antagonism of EP1, EP2, or EP4 (**Table 2**) attenuates multiple pain modalities, highlighting their pro-nociceptive activity (351–354). In contrast, activation of EP3 counteracts PGE_2_-induced sensitization (355). This antinociceptive effect is attributed to EP3 coupling to G_i_, leading to inhibition of cAMP production (356).

At the cellular level, PGE_2_ promotes nociceptor sensitization primarily through PKA- and PKC-dependent signaling pathways (357, 358). These pathways enhance sodium currents (359), suppress potassium currents (360, 361), and activate calcium channels (362), collectively increasing neuronal excitability. In the spinal cord, inflammation-induced upregulation of COX-2 in the dorsal horn further amplifies PGE_2_ signaling. Here, PGE_2_ inhibits the glycine receptor (363), thereby reducing inhibitory synaptic transmission onto excitatory interneurons and facilitating central sensitization.

PGE_2_ is as an active regulator of EGFR signaling, particularly through transactivation of EGFR by engaging both cell-surface and intracellular EP receptors (**Table 5**) (47, 364–367), with G coupling determining downstream outcomes. Unlike cell surface EP receptor signaling, which involves cAMP activation, nuclear EP receptors signal through a c-Src dependent mechanism to activate EGFR and downstream MAPK pathway (47). At the cell surface, EP receptors activate PKA and subsequent phosphorylation of intracellular molecules to allow surface trafficking of EGFR ligands (364). Once on the cell surface, EGFR pro-ligands are cleaved by MMPs to their active form.

**Table 5:** EGFR-PGE_2_ crosstalk in inflammation.

Cell type	Mode of EGFR transactivation	Key findings	Reference(s)
Human Renal Cells	src-mediated EGFR phosphorylation	↑ RAR-β, ↑HIF-1α	(47, 365)
Rat Gastric Epithelial Cells	Metalloproteinase-mediated HB-EGF shedding	↑COX-2	(373)
Rat Intestinal Epithelial Cells	Metalloproteinase-mediated EGFR ligand shedding	↑COX-2	(376)
Human Bronchial Epithelial Cells	MMP mediated EGFR ligand shedding	↑COX-2	(371)
Rat Vascular Smooth Muscle Cells	src mediated EGFR phosphorylation	↑COX-2	(368)
Brain Microvascular Endothelial Cells	src-mediated EGFR phosphorylation	↑COX-2	(372)
Human Cardiac Fibroblasts	Metalloproteinase-mediated HB-EGF shedding	↑COX-2→Apoptosis	(377)
Spinal Cord Astrocytes	src-mediated EGFR phosphorylation	↑PGE_2_	(114, 379)
Pulmonary Epithelial Cells	src-mediated EGFR phosphorylation	↑COX-2, ↑mPGES1	(374)
Renal Medullary Cells	Metalloproteinase-mediated TGFα shedding	↑COX-2→cell survival	(369)
Human lung fibroblasts	src-mediated EGFR phosphorylation	↑MIF, ↑COX-2, ↑IL-6, ↑MMP2	(375)
Rat Cerebella Astrocytes	Not determined	Protection against inflammatory injury	(367)
Human Synovial Fibroblasts	Metalloproteinase-mediated HB-EGF shedding	↑PGE_2_	(196)

EGFR transactivation by other GPCRs induces PGE_2_ production and MMPs are required to facilitate EGFR ligand shedding. This mechanism has been observed across vascular smooth muscle cells (368), synovial fibroblasts (196) and renal medullary cells (**Table 5**) (369). Similar EGFR ligand-dependent production of PGE_2_ is observed in gastric epithelial cells (370), bronchial epithelial cells (371), brain microvascular endothelial cells (372), gastric mucosal epithelial cells (373) and human lung epithelial/fibroblast cells (374, 375). In these cellular systems, multiple inflammatory stimuli, including PAR-1 (373), PAR-2 (376), LPA (371), sphingosine-1-phosphate (377), ATP (114, 378–380), endothelin-1 (372), ROS (381), and silica nanoparticles (381) require EGFR activation to induce COX-2 expression and subsequent PGE_2_ release. For instance, spinal cord injury or peripheral nerve injury releases nucleotides, such as ATP (114). Binding of these nucleotides to their receptors transactivates EGFR followed by PGE_2_ production (114, 379) which activates astrocytes and impairs neuronal function. However, these deleterious effect might depend on the EP receptor involved as uridine triphosphate (UTP) transactivation of EGFR was shown to activate EP3 receptor which inhibited the effects of elevated PGE_2_ in astrocytes (367).

These studies demonstrate that during tissue injury, high levels of PGE_2_ are attained through EGFR transactivation by EP receptors and other GPCRs. This bidirectional relationship forms a self-reinforcing inflammatory circuit that regulates cytokine production and pain sensitization which could explain why EGFR inhibition suppresses inflammation and pain.

### 4.7 EGFR and opioid receptors

Crosstalk between opioid receptors and receptor tyrosine kinases such as EGFR represents an important mechanism through which opioid signaling regulates intracellular pathways involved in neural plasticity and survival. Preclinical studies indicate that activation of μ-, δ-, and κ-opioid receptors can lead to transactivation of EGFR. One of the most widely characterized mechanisms of opioid-induced EGFR activation involves MMP-dependent cleavage of membrane bound EGFR ligands. Activation of the μ-opioid receptor (MOR) stimulates MMP activity, resulting in the release of endogenous ligands that subsequently activate EGFR and promote ERK phosphorylation (382). A similar mechanism has been observed for δ-opioid receptor (DOR) signaling, where receptor activation stimulates the release of HB-EGF and triggers EGFR-dependent activation of downstream ERK and Akt signaling cascades (60).

#### 4.7.1 Molecular mechanisms of EGFR-opioid receptor crosstalk

**Figure 4:**
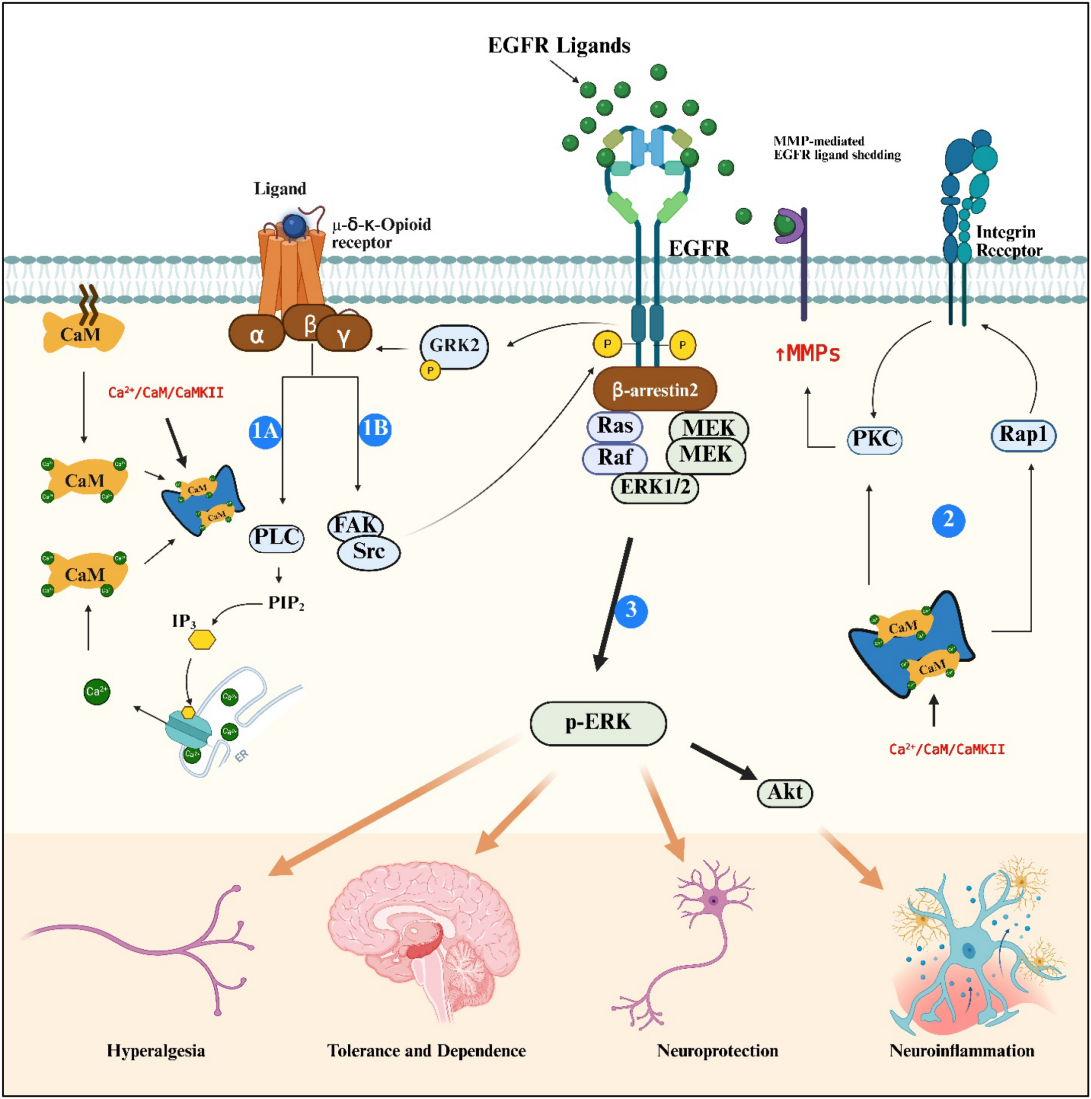
Crosstalk between opioid receptors and EGFR signaling pathways. Upon ligand binding, opioid receptors activate heterotrimeric G proteins (α, β, γ), initiating multiple downstream signaling cascades. (1A) Activation of phospholipase C (PLC) leads to hydrolysis of PIP_2_ into IP_3_, resulting in Ca^2+^ release from the endoplasmic reticulum (ER). (2) The resulting Ca^2+^/CaM/CaMKII stimulates protein kinase C (PKC), which promotes the expression of matrix metalloproteinases (MMPs) and subsequent shedding of EGFR ligands. Ca^2+^/CaM/CaMKII signaling also activates integrin receptors through Rap1, further stimulating PKC and enhancing MMP expression. (1B) In parallel, opioid receptor signaling engages focal adhesion kinase (FAK) and Src, which directly phosphorylate EGFR and promote downstream ERK activation. Activated EGFR can also phosphorylate G protein-coupled receptor kinase 2 (GRK2), promoting opioid receptor internalization. (3) These signaling events converge on ERK phosphorylation (p-ERK), a key mediator that regulates downstream pathways, including Akt signaling, contributing to hyperalgesia, opioid tolerance and dependence, neuroprotection, and neuroinflammation. Created in https://BioRender.com.

EGFR transactivation by opioid receptors is partly mediated by the calcium sensor, calmodulin (CaM). Under physiological condition, CaM is bound to the third intracellular loop of the opioid receptor and to the cell membrane (383) which reduces basal activity of the receptor and inhibits MMP activation (384). Upon morphine binding, CaM dissociates from the receptor and cell membrane triggering increased CaM in the cytosol and other cellular compartments where CaM can activate Ras-Raf complex (385, 386) and PLC to facilitate ERK phosphorylation via EGFR transaction (**Figure 4**) (384, 387). Notably, morphine treatment of striatal slices increases cytosolic and intracellular CaM content (388, 389) while mutant MOR does not bind CaM which limits the amount of CaM in the cytosol (383) as well as the levels of EGFR and ERK phosphorylation (384).

Other signaling molecules such as Src kinases and focal adhesion kinases (FAK) (390) interact with the EGFR when opioid receptors are activated, where src kinase can directly phosphorylate EGFR and activate it without ligand binding (**Figure 4**). Notably, a phosphopeptide targeting the interaction of src, FAK and EGFR prevented the prolongation of PGE_2_-induced hyperalgesia but partially reduced opioid induced hyperalgesia (OIH) indicating the presence of alternative pathways downstream the opioid receptors (382). For example, DOR activation has been shown to stimulate integrin-dependent signaling, which in turn promotes EGFR and ERK1/2 activation. In this pathway, opioid receptor activation induces PLC-dependent integrin activation and PKC-δ signaling (**Figure 4**) resulting in EGFR-transactivation (391). Scaffolding proteins such as β-arrestins also regulate the crosstalk between opioid receptors and EGFR. β-Arrestin2 can be recruited to the transactivated EGFR complexes and act as a scaffold for ERK signaling (**Figure 4**), enabling ligand-specific activation of downstream pathways following DOR stimulation (387). In addition, G protein-coupled receptor kinase 2 (GRK2) plays a role in bidirectional crosstalk between these receptors (**Figure 4**). For instance, EGFR activation can phosphorylate GRK2 and induce opioid receptor internalization (392, 393). Interestingly, EGFR transactivation is not always required for opioid-mediated ERK activation. In some cellular contexts, opioid receptors can switch to alternative receptor tyrosine kinase systems. Studies have shown that when EGFR signaling is downregulated, opioid receptor activation can stimulate ERK signaling through the insulin-like growth factor-1 receptor (IGF-1R), demonstrating redundancy and adaptability in opioid-RTK pathways (394).

Collectively, these studies highlight that opioid receptor-mediated EGFR transactivation is a complex and multifaceted process involving multiple signaling intermediates, including MMPs, Ca^2+^/CaM/CaMKII, Src, integrins, β-arrestins, and GRKs, as well as dynamic interactions with alternative receptor tyrosine kinase pathways.

#### 4.7.2 Pathological implications of EGFR-opioid receptor interactions

EGFR-opioid receptor interaction plays an important role in several pathological processes associated with chronic opioid exposure and neurological disorders. One major consequence of this signaling interaction is its contribution to opioid-induced hyperalgesia (OIH) and nociceptor sensitization. Repeated activation of MOR induces a form of neuroplasticity known as hyperalgesic priming, characterized by enhanced and prolonged sensitivity to inflammatory mediators such as PGE_2_ (382). Hence, pharmacological inhibition of EGFR prevents the prolongation of PGE_2_-induced hyperalgesia following opioid exposure (382). EGFR signaling is also implicated in the development of opioid tolerance and dependence. Chronic morphine exposure enhances EGFR phosphorylation and promotes the redistribution of MOR and EGFR within lipid raft microdomains in neurons, facilitating signaling interactions that lead to adenylyl cyclase superactivation, a biochemical hallmark of opioid tolerance (395). Persistent activation of ERK signaling by morphine has been attributed to sustained src activation and degradation of the ubiquitin ligase c-Cbl, which normally promotes EGFR downregulation (396). The loss of c-Cbl-mediated EGFR degradation leads to prolonged EGFR signaling and sustained MAPK activation (396).

Opioid-EGFR interaction also influences neuroplasticity and synaptic remodeling. In astrocytes, chronic MOR activation reduces the expression of thrombospondin proteins, which are extracellular matrix molecules that promote synapse formation and neurite outgrowth (397, 398). These changes are mediated by ERK signaling downstream of EGFR and may contribute to structural neuronal adaptations associated with chronic opioid exposure and addiction. Although many studies highlight detrimental consequences of EGFR-opioid interactions, this signaling axis may also exert neuroprotective effects under certain conditions. Activation of DORs has been shown to protect against cerebral ischemia in experimental models by stimulating EGFR-dependent signaling pathways. This process involves MMP-mediated release of EGFR ligands and activation of ERK and Akt, which ultimately reduces neuronal apoptosis and infarct size following ischemic injury (60).

#### 4.7.3 Therapeutic implications of EGFR-opioid receptor crosstalk

The growing understanding of EGFR-opioid receptor interaction has important implications for the development of new therapeutic strategies aimed at improving opioid analgesia while minimizing adverse effects. First, pharmacological targeting of EGFR signaling pathways may provide a strategy to reduce opioid-induced hyperalgesia and tolerance. EGFR inhibitors such as gefitinib inhibit morphine tolerance in rodents (70) and disrupt EGFR-dependent signaling cascades in nociceptors (382). In second, inhibition of upstream signaling components, including MMPs, may represent alternative strategies to modulate EGFR-opioid crosstalk. In the context of postoperative pain, EGFR-opioid crosstalk could be targeted with short-acting opioid agonists that selectively activate analgesic signaling pathways while avoiding EGFR-mediated mechanisms associated with tolerance and hyperalgesia. Such ligands could potentially maintain analgesic efficacy while preventing tolerance and dependence.

Increase of cytosol ca^2+^/calmodulin concentration activates the ca^2+^/calmodulin-dependent protein kinase II (CaMKII) which interacts with numerous synaptic proteins (399). CaMKII colocalize with MOR in the superficial dorsal horn and nociceptors (400, 401) and its inhibition prevents or reverses morphine tolerance, dependence and OIH in rodents (402, 403). However, preclinical CaMKII inhibitors have several off targets (402, 404, 405) which limits their use for clinical investigations. Optimizing these compounds could offer an avenue for combining opioid treatment with CaMKII inhibitors to prevent tolerance and dependence.

EGFR transactivation represents a critical mechanism linking opioid receptor signaling with growth factor receptor pathways. Multiple molecular mechanisms, including MMP-dependent EGFR ligand shedding, calmodulin-dependent signaling, src kinase activation, integrin-mediated pathways, and β-arrestin scaffolding, contribute to the ability of opioid receptors to activate EGFR and downstream MAPK signaling. While these interactions enable opioid receptors to regulate cellular processes such as neuronal plasticity and survival, they also contribute to adverse effects associated with chronic opioid use. Understanding the molecular basis of EGFR-opioid receptor crosstalk may facilitate the development of novel therapeutic strategies aimed at improving opioid analgesia while minimizing adverse effects.

## 5 Safety and tolerability of EGFR tyrosine kinase inhibitors

EGFR tyrosine kinase inhibitors (EGFR-TKIs) represent an important class of targeted anticancer therapies designed to inhibit signaling mediated by the EGFR. These inhibitors act by competing with ATP for binding to the cytoplasmic kinase domain of the receptor, thereby preventing receptor autophosphorylation and subsequent activation of downstream signaling pathways (97). Through this mechanism, EGFR-TKIs have significantly improved patient outcomes in several malignancies, particularly cancers driven by activating EGFR mutations. Despite their clinical benefits, these therapies are associated with specific adverse events that may influence treatment adherence and patient quality of life.

In general, EGFR-TKIs are considered safer and more tolerable than conventional cytotoxic chemotherapy (406). Evidence from clinical trials and meta-analyses indicates that severe toxicities are rare and differ between drugs (407–411). The most reported adverse effects are dermatologic and gastrointestinal in nature, including skin rash and diarrhea (412–416), but are effectively managed with topical corticosteroids (417) and antidiarrheal medications (418, 419), respectively. These toxicities arise primarily because EGFR signaling is essential for the maintenance and repair of normal epithelial tissues, particularly in the skin and gastrointestinal tract. Consequently, inhibition of EGFR in healthy tissues can disrupt normal cellular processes, leading to these characteristic adverse effects (402–423). Although most adverse events are mild to moderate and can be effectively managed, more severe toxicities require dose reduction or discontinuation of therapy (424, 425).

Another important factor influencing the safety profile of EGFR-TKIs is their potential for off-target activity. Receptor tyrosine kinase inhibitors may exhibit promiscuous binding due to the highly conserved nature of the ATP-binding motif shared among numerous protein kinases and other ATP-binding cassette (ABC)-containing proteins, such as cellular transporters (426). At higher concentrations, this lack of selectivity can result in nonspecific inhibition of additional kinases or transporters, thereby contributing to unintended biological effects. Such off-target interactions have been associated with toxicities affecting multiple organ systems, including cardiovascular complications (427).

Importantly, recent preclinical research exploring the repurposing of EGFR-TKIs for pain management suggests that therapeutic effects may be achieved at substantially lower doses than those used in oncology. In several preclinical pain models, subclinical doses of EGFR-TKIs were sufficient to produce analgesic effects (26, 32, 70, 117) without inducing the toxicity typically observed at higher anticancer doses. These findings suggest that, if EGFR-TKIs are repurposed for the treatment of pain, lower dosing strategies may reduce the risk of adverse effects while maintaining therapeutic efficacy. Consequently, dose optimization may play a critical role in improving the safety and tolerability of these agents in non-oncologic applications.

## 6 Future directions and therapeutic optimization

### 6.1 EGFR inhibition in postoperative pain

Despite emerging evidence implicating EGFR signaling in nociceptive processing, its role in postoperative pain remains poorly understood. Future studies should focus on establishing a direct causal relationship between inflammatory signaling and EGFR activation following surgical injury. Defining the network of inflammatory mediators that interact with EGFR during postoperative pain may help clarify how inflammatory signals converge on this pathway to promote central sensitization.

Although EGFR inhibition has been reported not to suppress the anti-inflammatory cytokine IL-10, it remains unclear whether other anti-inflammatory mediators are preserved or differentially regulated. Determining how these cytokine profiles influence neuronal excitability and synaptic plasticity will be essential for understanding the broader immunomodulatory effects of EGFR inhibition. Furthermore, the effects of EGFR inhibition on PGE_2_ production within CNS regions remain poorly characterized, despite the well-established role of PGE_2_ in pain. Emerging evidence also suggests that EGFR signaling in astrocytes and microglia (37, 56, 428) may represent an additional mechanism through which inflammatory signals regulate neuronal excitability. Investigating EGFR-mediated signaling in glial cells may therefore provide further insight into how neuroimmune interactions contribute to synaptic plasticity and central sensitization in postoperative pain.

Progress in this research area may be limited by the heavy reliance on the rodent incision model, which remains the most widely used preclinical assay for evaluating analgesic compounds. Although this model reliably captures acute postoperative pain behaviors, it does not fully recapitulate the mechanisms underlying chronic postoperative pain. Incorporating more translational models that better mimic the transition from acute to persistent postoperative pain would enable researchers to determine at which stage EGFR signaling contributes to the development of long-term pain states and whether EGFR inhibition could prevent the transition from acute to chronic postoperative pain.

### 6.2 Biomarker strategies for tracking EGFR and its ligands

Several approaches have been used to monitor EGFR and its ligands in biological samples including blood, cerebral spinal fluid (CSF), brain homogenates, cortical neurons, nociceptors, glial cells, DRG, dorsal horn, cell culture supernatants (130) and serum. Many of these strategies focus on assessing receptor activation by measuring the phosphorylation of EGFR and its downstream mitogen-activated protein kinases (MAPKs) using western blotting (28, 37, 49, 50, 54, 55, 68, 117).

Other studies have quantified EGFR ligands using a range of biochemical and molecular techniques. For example, chemiluminescence-based assays and ELISA have been used to measure EREG levels in intervertebral discs (429) and HB-EGF in CSF and brain homogenates (60), respectively. Multiplex bead-based assays have been used to quantify AREG, BTC, and TGFα in human serum samples (126). Reverse transcription quantitative PCR (RT-qPCR) has been employed to quantify EREG transcripts in blood samples (26). In addition, next-generation sequencing has been utilized to genotype single nucleotide polymorphisms (SNPs) in EGFR and its ligands in a cohort of patients with orofacial pain (33). Finally, techniques such as RNAscope *in situ* hybridization (111, 430), immunohistochemistry (IHC), and immunofluorescence microscopy have been applied to localize and characterize EGFR expression in neuronal and non-neuronal tissues (26, 71, 431).

### 6.3 EGFR combination therapy

Combination therapies targeting EGFR signaling alongside conventional analgesics or anti-inflammatory agents may enhance analgesic efficacy while reducing the doses required for individual drugs. Pharmacokinetic studies demonstrate clinically relevant interactions between EGFR-TKIs and commonly used analgesics. Co-administration of the EGFR inhibitor lapatinib with paracetamol has been shown to increase lapatinib exposure while reducing paracetamol exposure and increasing paracetamol sulphonation, potentially elevating the risk of toxicity (432). Similarly, sunitinib significantly alters the plasma exposure of intravenously administered paracetamol and its major metabolite, paracetamol glucuronide (433). Additional preclinical studies in mice reveal sex-dependent pharmacokinetic interactions between sunitinib and paracetamol, suggesting that EGFR-TKIs can influence analgesic metabolism and tissue distribution (434). These findings underscore the importance of considering pharmacokinetic interactions when combining EGFR inhibitors with commonly used analgesics in clinical contexts.

Interactions between EGFR-targeted therapies and NSAIDs further highlight the complexity of combination strategies. Preclinical studies examining sunitinib in combination with NSAIDs such as diclofenac, mefenamic acid, and ibuprofen demonstrate sex-divergent effects on the tissue uptake and distribution of the kinase inhibitor in mice (434). Notably, ibuprofen co-administration alters the pharmacokinetics and tissue distribution of sunitinib in multiple organs, including the brain, liver, and kidney (435). Given the central role of the nervous system in nociceptive processing, these findings suggest that NSAIDs may influence the distribution and pharmacological activity of EGFR-TKIs in tissues relevant to pain signaling. Furthermore, clinical observations show that NSAIDs may reduce the incidence of EGFR-TKI-associated skin rash, suggesting that NSAIDs can modify EGFR-driven biological responses (436).

Mechanistic evidence further supports the therapeutic potential of combined EGFR and anti-inflammatory drugs. Preclinical studies indicate that NSAIDs can enhance the efficacy of EGFR inhibition by overcoming resistance mechanisms mediated through PI3K signaling pathways (437). Clinical oncology studies have also explored dual targeting approaches, such as the combination of the EGFR-TKI gefitinib with the COX-2 inhibitor celecoxib (438), demonstrating the feasibility of simultaneously inhibiting EGFR and inflammatory signaling pathways.

Although these studies were conducted in cancer settings, they provide an important conceptual framework for pain therapy. Collectively, these findings suggest that EGFR-based combination therapies with anti-inflammatory agents is feasible and worth exploring.

### 6.4 Targeted drug delivery systems in the management of postoperative pain

EGFR-TKIs are administered orally, however, poor solubility, extensive plasm binding and permeability limits their oral bioavailability (439, 440). Advances in targeted drug delivery provide an avenue for localized delivery strategies that concentrate EGFR inhibitors in the affected tissues. In postoperative pain settings, advanced delivery platforms, such as nanoparticle carriers, liposomal formulations, or hydrogel-based depots, could theoretically enable targeted delivery of EGFR-TKIs to injured peripheral tissues, DRGs, or spinal cord regions involved in pain processing. Such approaches may enhance analgesic efficacy while minimizing systemic exposure and adverse effects associated with systemic EGFR inhibition. Although these delivery strategies have primarily been developed in oncology (441–448), their ability to achieve sustained and localized drug release suggests strong translational potential for postoperative pain management.

### 6.5 EGFR signaling as a diagnostic and prognostic marker in chronic inflammatory pain

Chronic inflammatory pain is characterized by persistent nociceptor sensitization and neuro-immune interactions that sustain pathological pain signaling. Identifying reliable biomarkers capable of predicting disease onset, severity, and treatment response remains a major challenge in pain medicine. The EGFR signaling pathway may serve as a valuable source of diagnostic and prognostic biomarkers in chronic inflammatory pain. Because EGFR signaling becomes dysregulated in inflammatory states and directly modulates nociceptor activity, components of this pathway, including receptor activation levels, circulating ligands, and genetic variants, can be potential biomarkers for chronic inflammatory pain.

#### 6.5.1 EGFR activation as a diagnostic marker

One potential strategy for predicting pain outcomes involves quantifying EGFR expression and phosphorylation levels in pain-related tissues such as DRGs, nociceptors, and spinal cord. From a clinical perspective, measuring phosphorylated EGFR (p-EGFR) in tissue biopsies or circulating extracellular vesicles could serve as a diagnostic biomarker indicating active inflammatory pain signaling (449). In addition, EGFR ligands themselves may serve as measurable biomarkers of inflammatory pain. Many of these ligands are produced by immune cells, epithelial cells, and neurons in response to tissue injury and inflammation. Because EGFR ligands can be released into extracellular fluids, circulating concentrations of ligands such as EREG, AREG, or HB-EGF in plasma or serum could potentially serve as non-invasive biomarkers reflecting inflammatory pain activity (126, 130). Monitoring changes in ligand levels may therefore provide insight into disease progression or treatment response.

Genetic studies further support the potential of the EGFR pathway as a biomarker for chronic pain susceptibility. Genome-wide association studies and candidate gene analyses have identified genetic variants in EGFR and its ligand EREG that are associated with increased risk of chronic pain conditions (26). For example, variants in EGFR and EREG genes have been linked to the development of temporomandibular disorder (TMD), a chronic pain condition characterized by persistent inflammation and nociceptor sensitization (26). These findings suggest that genetic polymorphisms affecting EGFR signaling may predispose individuals to chronic pain, potentially through altered receptor signaling or ligand expression. Genetic screening for EGFR pathway variants could therefore serve as a predictive biomarker for identifying individuals at higher risk of developing chronic inflammatory pain.

#### 6.5.2 Prognostic value of EGFR signaling in pain progression

EGFR signaling components may also serve as prognostic biomarkers capable of predicting disease progression and therapeutic outcomes. Persistent EGFR activation promotes long-term neuronal sensitization through activation of intracellular pathways such as mTOR and MAPK, which regulate protein synthesis, synaptic plasticity, and inflammatory mediator production. These mechanisms contribute to the transition from acute to chronic pain. Therefore, elevated EGFR activity or increased levels of its ligands may indicate a greater likelihood of pain chronicity or severe disease progression.

Accumulating evidence indicates that the EGFR signaling pathway plays a critical role in the development and maintenance of chronic inflammatory pain. Components of this pathway, including receptor activation levels, circulating ligands such as EREG, and genetic variants in EGFR-related genes, represent promising diagnostic and prognostic biomarkers. By reflecting underlying mechanisms of nociceptor sensitization and neuro-immune interactions, EGFR pathway biomarkers may enable earlier detection of chronic pain states, improve risk prediction, and guide targeted therapeutic strategies.

Further clinical studies are required to validate these biomarkers in diverse pain populations and to establish standardized assays for their measurement.

## 7 Conclusion

Postoperative pain is driven by a complex and highly redundant network of inflammatory mediators. Early suppression of inflammation is essential for effective analgesia, yet targeting a single mediator often proves insufficient because parallel pathways compensate rapidly. The evidence reviewed here suggests that EGFR occupies a unique position within this network. Its activation promotes cytokine, chemokine, prostaglandin, and matrix metalloproteinase production as well as simultaneous engagement of signaling pathways that promote pain. Because EGFR inhibitors are widely used in oncology and have not demonstrated addictive properties or severe life-threatening adverse effects, repurposing these agents for postoperative pain represents a promising therapeutic strategy. Collectively, the available evidence justifies further investigation of EGFR blockade as a multifaceted intervention capable of attenuating both inflammatory and neuronal drivers of postoperative pain.
